# Supplementary material for: scMGCN: A Multi-View Graph Convolutional Network for Cell Type Identification in scRNA-seq Data
Source: Int J Mol Sci. 2024 Feb 13;25(4):2234. doi: 10.3390/ijms25042234 (PMC10889820; doi:10.3390/ijms25042234)
Supplement: Supplementary file 1 [file ijms-25-02234-s001.zip › supplementary materials.pdf]

International Journal of
Molecular Sciences

MDPI

Supplementary Materials: scMGCN: A Multi-View Graph Convolutional Network for Cell Type Identification in scRNA-seq Data

Hongmin Sun [1,†], Haowen Qu [1,†], Kaifu Duan [1] and Wei Du[1,*]

1. Experimental results on each dataset

1.1. Experimental results for the performance of multi-view fusion on each dataset

Figures S1 to S4 illustrates the comparative results of multi-view and single-view methods in single dataset, cross-species, and cross-platform experiments in terms of Accuracy and Macro-F1.

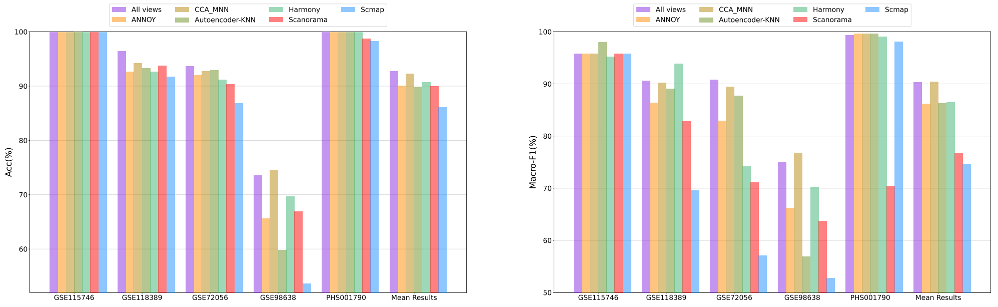

Figure S1. Comparison of multi-view and single-view methods in single dataset experiments w.r.t. Accuracy and Macro-F1.

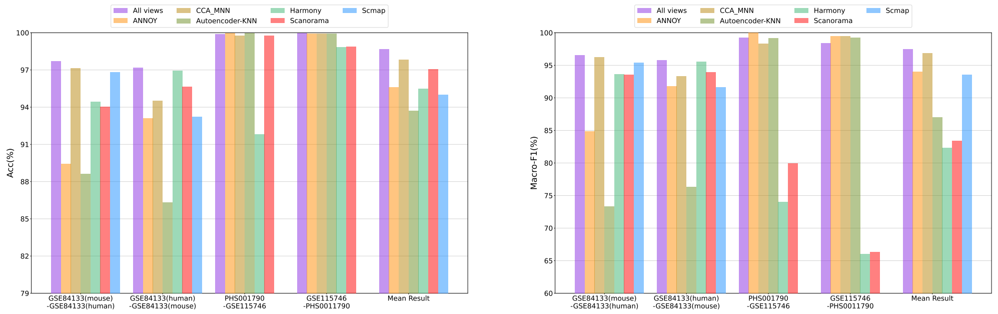

Figure S2. Comparison of multi-view and single-view methods in cross-species experiments w.r.t. Accuracy and Macro-F1.

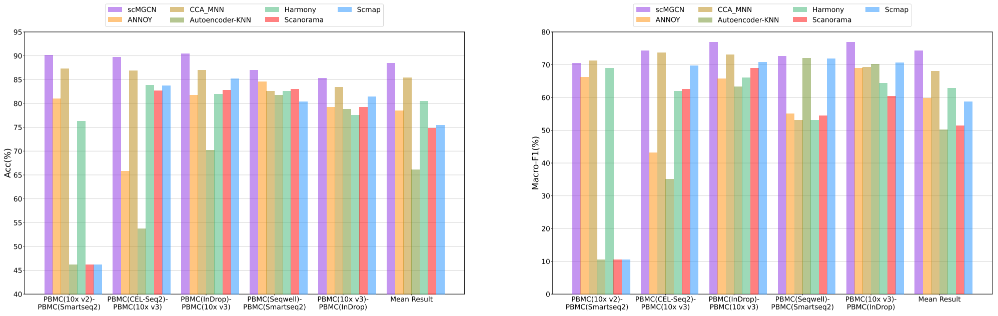

Figure S3. Comparison of multi-view method and single-view method in cross-platform(PBMC) experiments w.r.t. Accuracy and Macro-F1.

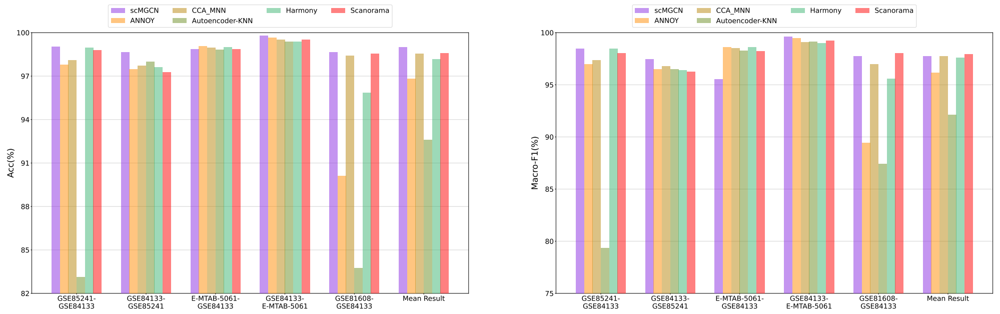

Figure S4. Comparison of multi-view method and single-view method in cross-platform(Pancreas) experiments w.r.t. Accuracy and Macro-F1.

1.2. Experimental results for the Performance of different network modules on each dataset

Figures S5 to S8 illustrates the comparative results of scMGCN and two other existing graph neural network-based multi-view fusion models in single dataset, cross-species, and cross-platform experiments in terms of Accuracy and Macro-F1.

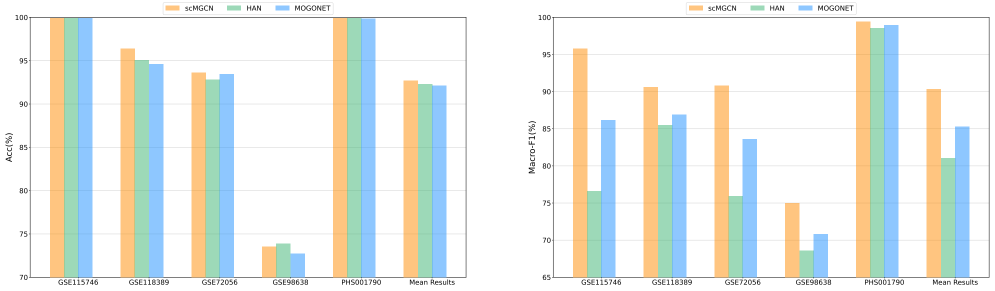

Figure S5. Comparison of scMGCN and two other existing graph neural network-based multi-view fusion models in single dataset experiments w.r.t. Accuracy and Macro-F1.

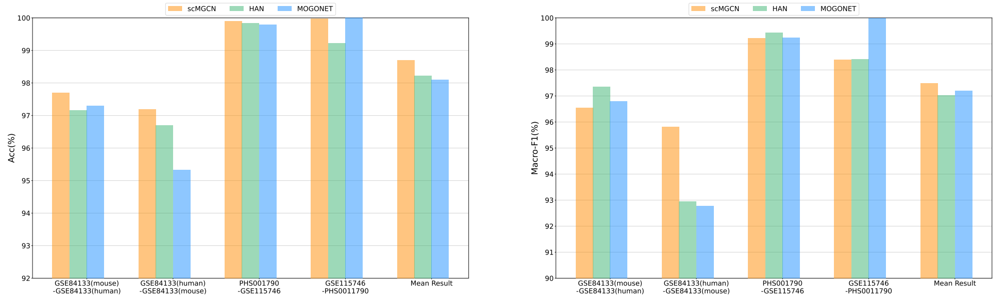

Figure S6. Comparison of scMGCN and two other existing graph neural network-based multi-view fusion models in cross-species experiments w.r.t. Accuracy and Macro-F1.

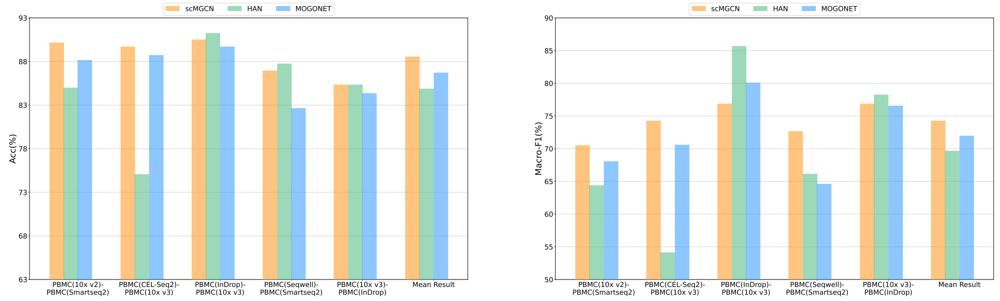

Figure S7. Comparison of scMGCN and two other existing graph neural network-based multi-view fusion models in cross-platform(PBMC) experiments w.r.t. Accuracy and Macro-F1.

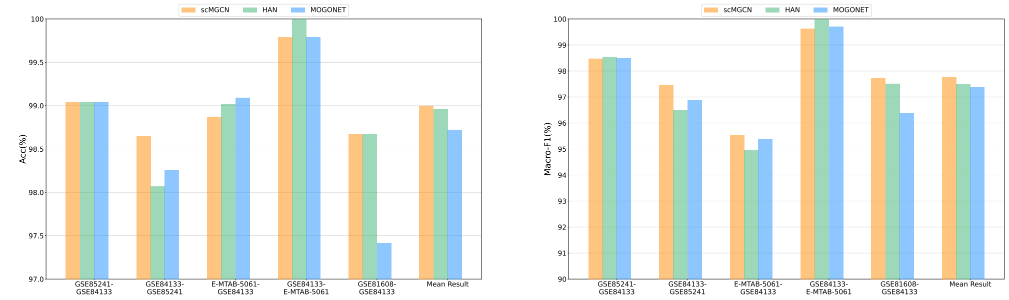

Figure S8. Comparison of scMGCN and two other existing graph neural network-based multi-view fusion models in cross-platform(Pancreas) experiments w.r.t. Accuracy and Macro-F1.

2. The details for graph construction

The main idea of ANNOY is to establish a binary tree structure for the data. First, two random nodes are selected to perform a k-means clustering with k=2. After selecting the two nodes as the clustering centers, the Euclidean distances between all nodes and the two centers are calculated. ANNOY sacrifices some query accuracy to achieve efficient search speed, making them very useful for querying large-scale high-dimensional data. Due to the efficiency improvement of ANNOY at the expense of partial query accuracy, the precision of query results may be affected.

The goal of CCA_MNN is to perform dimension reduction on two datasets, projecting high-dimensional reference and query datasets into a low-dimensional space through two linear transformations based on the datasets. CCA_MNN jointly utilizes multiple graph networks, enabling the capture of multiple levels of features in the data, thereby enhancing the richness of node representations. However, due to the need for CCA_MNN to process large-scale graph data, it may not achieve optimal results for small-scale datasets.

CellHarmony is an integration algorithm for multi-batch single-cell data, which mainly consists of two parts: maximizing clustering diversity and linear mixed model correction. CellHarmony can accurately match single-cell transcriptomes and calculate the differences in cell type-specific gene expression for dozens of cell populations. However, the performance of CellHarmony depends on the quality of the input data. If there are biases or errors in the data, it may affect the accuracy of the results.

Scanorama is a cell integration tool proposed based on image panorama stitching technology. It uses singular value decomposition to calculate the low-dimensional embedding of each cell's gene expression value. This method uses an extended quality control method for standardization, which can effectively remove systematic errors such as batch effects and improve data consistency and comparability. However, this method is sensitive to parameter settings.

Scmap is a tool for unsupervised cell mapping that can map query cells to clusters in the reference set as well as to cells in the reference set. However, it is sensitive to parameters and data quality, including single-cell sequencing depth, data normalization, batch effects, and more.

Autoencoder-KNN is a hybrid model that combines an autoencoder with a k-nearest neighbors (KNN) classifier. This method can both utilize the autoencoder to learn the inherent representation or features of the input data, thus better representing the data, and utilize the KNN classifier to vote on the category of the input data based on the categories of its k nearest neighbors, thereby improving classification accuracy. However, this method is sensitive to the size of input features, noise, and outliers.

3. The related code and datasets

The related code and datasets can be found on https://github.com/dawn-creator/scMGCN (accessed on 5 December 2023).
